# Curriculum Innovation: A Multicenter Feasibility Study of a Consensus-Based Brain Death Simulation Curriculum

**DOI:** 10.1212/NE9.0000000000200335

**Published:** 2026-07-02

**Authors:** Amjad Elmashala, Catherine S.W. Albin, Daniel S. Harrison, Lauren Koffman, Xin Zhou, Jamie Nicole LaBuzetta, Rachel B. Beekman, Stefanie P. Cappucci, Matthew Bevers, Hera Kamdar, Matthew T. Hoerth, Ariane Lewis, Sarah Wahlster, Justine Cormier, Jenna Ford, David Matthew Greer, Nicholas A. Morris, for CRESCENT Collaboration

**Affiliations:** 1 Department of Neurology, Rush University Medical Center, Chicago, IL;; 2 Department of Neurology, Emory University, Atlanta, GA;; 3 Boston University Chobanian and Avedisian School of Medicine, Boston Medical Center, MA;; 4 Department of Neurology, Temple University Hospital, Philadelphia, PA;; 5 Department of Neurosciences, University of California - San Diego, La Jolla, CA;; 6 Department of Neurology, Yale University, New Haven, CT;; 7 Department of Neurology, Mass General Brigham, Boston, MA;; 8 Department of Neurology, The Ohio State University, Columbus, OH;; 9 Department of Neurology, Mayo Clinic, Phoenix, AZ;; 10 NYU Langone Medical Center, New York, NY;; 11 Departments of Neurology, Neurological Surgery, and Anesthesiology, University of Washington, Seattle, WA;; 12 Ayer Neuroscience Institute, Hartford Hospital & University of Connecticut, Hartford, CT;; 13 Department of Neurology, Northwestern Feinberg School of Medicine, Chicago, IL; and; 14 Department of Neurology, Program in Trauma, University of Maryland School of Medicine, Baltimore, MD.

## Abstract

**Introduction and Problem Statement:**

Competency in brain death/death by neurologic criteria (BD/DNC) determination is an Accreditation Council for Graduate Medical Education (ACGME) milestone in neurology residency and neurocritical care fellowship; however, trainees have limited clinical exposure and receive inconsistent training across institutions. Simulation offers a standardized, safe environment to teach and assess BD/DNC, but multi-institutional implementation remains unstudied. The aim of this study was to evaluate the feasibility of implementing a consensus-based BD/DNC curriculum and to assess its impact on trainee confidence.

**Learning Objectives:**

By the end of the curriculum, trainees will be confident in assessing BD/DNC prerequisites, performing a complete neurologic examination, safely conducting and interpreting apnea testing, and accurately declaring BD/DNC.

**Methods and Curriculum Description:**

Through an iterative consensus process, 3 simulation cases addressing BD/DNC confounders were developed and implemented across high-fidelity and low-fidelity settings. Neurology residents and neurocritical care fellows were recruited by email from 6 academic centers in the United States. Participants each completed one case and received debriefing. Learning objectives were assessed using a 17-item, 5-point Likert pre/postsurvey assessing confidence across 4 BD/DNC domains: “Setup and Family Counseling,” “Prerequisites,” “Neurologic Examination,” and “Apnea Test and Declaration of BD/DNC.” Composite domain medians were compared using the Wilcoxon signed-rank test, and change scores (post–pre) were compared between fidelity groups using the Mann-Whitney *U* test.

**Results:**

Nineteen participants (9 female [47%]; predominantly PGY-4 trainees) were enrolled. Median [IQR] pre-post composite confidence scores improved significantly across all domains: “Setup and Family Counseling,” 4 [3.5–4] to 4.5 [4–5]; “Prerequisites,” 4 [4–5] to 4.5 [4–5]; “Neurologic Examination,” 4 [4–4] to 4.5 [4–5]; and “Apnea Test and Declaration of BD/DNC,” 3 [3–4] to 5 [4–5]. The effect sizes (Wilcoxon r) ranged from 0.79 to 0.88 across domains. There were no significant differences in the magnitude of improvement between high-fidelity and low-fidelity groups. After curriculum implementation, 13 of 19 participants (68%) self-assessed their ACGME BD/DNC milestone competency to be level 4 or 5.

**Discussion and Lessons Learned:**

This multi-institutional pilot demonstrated that a consensus-based BD/DNC simulation curriculum is feasible and improves trainee confidence despite limited previous clinical exposure. Key lessons include the feasibility of multicenter collaboration, standardizing core practices while accommodating local variability, and designing adaptable curricula that achieve similar outcomes across resource-diverse settings.

## Introduction and Problem Statement

The 2023 brain death/death by neurologic criteria (BD/DNC) consensus guidelines recommend that all clinicians performing BD/DNC examinations demonstrate competency; however, mechanisms to achieve and maintain competence were not specified.^1^ The Accreditation Council for Graduate Medical Education (ACGME) milestones, which are developmental, competency-based outcomes, advise that neurology residents and neurocritical care fellows should be able to properly perform BD/DNC evaluation by graduation.^2,3^ Despite these mandates, there are clear opportunities for improvement in BD/DNC education. In a survey of 68 physicians involved in BD/DNC determination, 24% reported never receiving training and only 25% reported completing all components of the brain death examination.^4^ These performance gaps may have clinical implications; on review, neurointensivists had concerns about practice deviations in 19% of declared BD/DNC cases referred to an organ procurement center.^5^ These data underscore an existing gap between guidelines, ACGME expectations, and real-world training and practice.

Several factors contribute to trainees' lack of preparedness to accurately and consistently determine BD/DNC. Clinical exposure is often limited, with many residents and fellows encountering fewer than 5 BD/DNC cases during training.^6^ Institutional protocols are also heterogeneous, with marked variation in prerequisites, examination procedures, and examiner qualifications,^7,8^ creating inconsistent training opportunities and experiences.

Simulation-based training offers a standardized, safe environment in which learners can practice the complete BD/DNC evaluation under uniform conditions while also navigating pitfalls and confounders. Although several single institutions have published BD/DNC,^9-12^ they are limited by (1) their single-center design and small sample size, which limit generalizability; (2) a lack of guiding theorectical and conceptual frameworks; and (3) insufficient validity evidence supporting their use as assessment tools. In response, there has been a call for standardized education and assessment of BD/DNC.^13^

To address these concerns, we convened a large, diverse group of simulation educators and BD/DNC experts to collaboratively develop a simulation-based mastery learning (SBML) curriculum for BD/DNC determination. SBML is grounded in behavioral, constructivist, and social cognitive learning theories.^14^ It incorporates baseline testing, clear learning objectives, deep engagement in educational activities with deliberate practice and real-time feedback, and a commitment to mastery through formative assessment to achieve a minimum passing score.^15^ This rigorous, practice-based approach to achieving documented competency is well suited to teaching BD/DNC determination because errors in this high-stakes evaluation may lead to an inaccurate determination of death. In this report, we share the consensus-based curriculum development, experience with implementation across 6 institutions, preliminary validity evidence, and pilot participant assessment data for a novel SBML curriculum. We also share our lessons learned—including challenges and solutions—in developing, deploying, and studying a multicenter educational innovation in BD/DNC teaching and assessment.

## Learner Objectives

By the end of the program, participants will gain confidence in their ability to

1. Evaluate prerequisites for BD/DNC determination.
2. Perform a complete and accurate neurologic examination to determine BD/DNC.
3. Safely perform apnea testing, including assessing eligibility, identifying when to abort the test, and interpreting results.
4. Correctly declare death by neurologic criteria and correctly document time of death.

## Methods and Curriculum Description

In this report, we first describe our process for developing the BD/DNC curriculum and gathering preliminary validity evidence as an assessment tool using the Messick framework, focusing on content, response process, and internal structure.^16^ Our goal (the construct) is “BD/DNC determination,” and the interpretation is that “participants can demonstrate knowledge of the prerequisites for BD/DNC determination, perform the complete clinical examination and apnea testing, determine the need for ancillary testing, and correctly declare time of death.” Ultimately, these cases are designed to be part of a SBML curriculum, which will evaluate neurology residents' and neurocritical care fellows' competency to perform BD/DNC determination.

We then describe the process of piloting the curriculum nationally and the collection of Kirkpatrick Level 1 data from the pilot participants across multiple centers. We also evaluated adaptation of the curriculum in high-fidelity and low-fidelity simulation settings.

### Curriculum Design and Iterative Improvement Process

#### Case Development

Because there are a variety of potential pitfalls and confounders to BD/DNC determination that trainees should be aware of, we developed 3 separate cases to broadly represent the diversity of challenges encountered in BD/DNC determination (eAppendix 1). Each of the 3 BD/DNC cases begins with a clinical vignette describing the patient's presentation and hospital course. In each, the primary team requests the participant to evaluate for BD/DNC. Pitfalls or confounders vary by case. These confounders fall within the domains of prerequisites for performing the examination, the clinical examination, apnea testing, and/or the need for ancillary testing, as listed in Table 1.

**Table 1:** Key Pitfalls and Confounders in the Prerequisites, Clinical Examination, and Requirement for the Apnea Test for Brain Death/Death by Neurologic Criteria (BD/DNC) Cases (A, B, and C)

	Case A	Case B	Case C
Pitfalls/confounders in prerequisites	• Repeat imaging to document presence of catastrophic, permanent, neurologic injury • Morphine administration on MAR review • Hypothermia	• Need to wait >24 h to assess for permanency of brain injury • Urine drug screen positive for fentanyl	• Repeat imaging to document presence of catastrophic, permanent, neurologic injury
	Mean arterial blood pressure augmentation to >75 mmHg		
Pitfalls/confounders in clinical examination	Need to adjust head of bed before checking oculovestibular reflex	Left eye swelling from initial injury, precluding ability to test pupillary and corneal reflexes on that eye	Right lower extremity triple flexion (through video)
Pitfalls/confounders in the apnea test	• High ventilator settings • Respiratory acidosis • Hypotension and hypoxia	• Respiratory acidosis	• Respiratory alkalosis
Ancillary test	Not required	Required	Not required

#### Content Validity Evidence in Case Development

Content validity evidence refers to the degree to which the assessment, including the scenario and response options, accurately reflects the construct it intends to measure.^17^ To establish content validity, the cases were written by several authors based on real-life cases that could highlight pitfalls and/or confounders in the BD/DNC determination examination. Special attention was paid to emphasize the changes between the updated 2023 BD/DNC guidelines and previous iterations of the guidelines, specifically the minimum mean arterial blood pressure, 24 hours of observation period after cardiac arrest, required neuroimaging evidence of catastrophic supratentorial injury in patients with primary posterior fossa injury, mandatory waiting periods after hypothermia, and arterial blood gas targets before performing apnea testing.^18^ The case content was reviewed and edited by experts in BD/DNC determination and multiple neurointensivists and educators with simulation expertise and BD/DNC evaluation experience. The cases were iteratively refined over several virtual meetings of educators with an interest in BD/DNC determination from 23 institutions across the United States from November 2024 to February 2025, before piloting the simulation at the first center. Evidence for content validity was also established by maintaining a high degree of fidelity in how the “patient chart” material was presented. For example, all simulated patient charts included a vital signs flowchart, medication administration review (MAR) panel, laboratory results panel, and scrollable radiography that mimicked the appearance of an electronic health record workspace. This allowed the participant to access the data in a format that mirrored real-life clinical experience.

#### Content Validity Evidence for Assessment (Critical Action Checklist)

Authors of the 2023 Adult and Pediatric BD/DNC Guideline and World Brain Death Project developed a critical action checklist and a minimal passing standard using a modified Delphi process and the Angoff standard-setting process. That process is reported elsewhere.^19^ During pilot testing, we assessed usability of the critical action checklist. Pilot testing informed interpretation and scoring of the critical action checklist as described in the following section.

#### Response Process Validity Evidence in Pilot Testing

Within the Messick framework, response process contributes to validity by confirming that the action of learners and raters align with the intended construct. To ensure that all participants understood the rules of engagement, we developed a standardized prebriefing (eAppendix 2). The prebriefing script was modified based on participant performance and feedback. For instance, a few pilot participants simply asked for the examination findings rather than directly examining the simulated patient. To correct this, we added the following to the prebrief: “Since we will be assessing your exam skills, please perform the exam exactly as you would on a real patient (and do not simply ask for exam findings).” Furthermore, pilot case video recordings were reviewed during monthly investigator meetings held from March 2025 to July 2025 to ensure standardized case implementation.

#### Internal Structure Validity Evidence in Pilot Testing

Internal structure validity evidence evaluates the relationships of individual assessment items with each other and with the overarching construct.^17^ Selected, full-length recordings of the pilot cases were reviewed at the monthly investigator meetings to calibrate rater scoring by consensus; in total, 6 recordings were reviewed, 2 of each case. Inter-rater reliability between in-person assessors and blind raters will be evaluated as part of the ongoing SBML curriculum.

#### Pre–/Post–Self-Assessment Surveys

Pre-/postsimulation surveys assessed participants' previous experience in BD/DNC determination, their self-reported confidence, and their reactions to the training. The survey included 17 items assessing confidence in performing BD/DNC tasks, mapped to the learning objectives (eTable 1). Items were rated on a 5-point Likert scale (1 = strongly disagree, 5 = strongly agree). The postsurvey further included a self-assessment item on BD/DNC determination based on the ACGME milestones (eTable 2).^2^ As part of the response process, pilot trainees were asked to comment on any question or self-rating that was unclear to them or could be misinterpreted.

### Pilot Testing and Data Collection

After curriculum design, we sought to demonstrate the feasibility of implementing the consensus BD/DNC simulation curriculum across a wide variety of low-fidelity and high-fidelity simulation environments. As a secondary objective, we sought to evaluate trainee-reported confidence in BD/DNC, measured using pre/postsimulation surveys.

#### Pilot Testing

**Figure 1:**
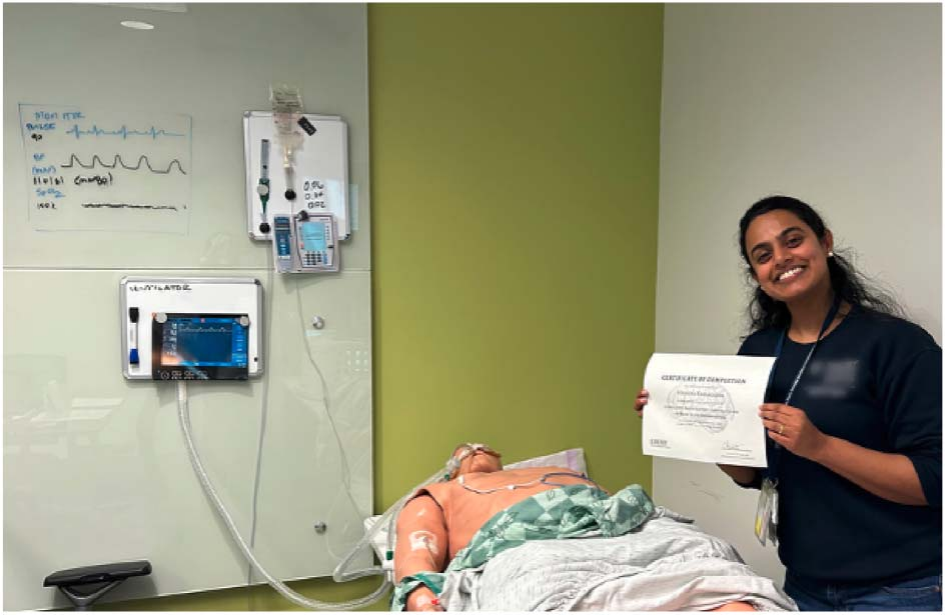
Low-Fidelity Brain Death/Death by Neurologic Criteria Simulation A trainee completing a brain death simulation session in a low-fidelity setting conducted in a classroom environment, using a noninteractive manikin. The available manikin could not react, but participants could still open the manikin's eyes and demonstrate how to check for a pupillary light and corneal reflex. The manikin was intubated, and trainees were expected to demonstrate the apnea testing by advancing an insufflation catheter to the level of the carina. Printout versions of the programmable pump and ventilator screen could be changed according to the action of the trainee. Either an iPad “monitor” or the white board was used to display vital signs and was updated by the embedded participant as the case unfolded. Trainees were still expected to review the ventilator settings and review the medications being titrated.

These 3 simulation cases were piloted at 6 neurology and neurocritical care programs: University of Maryland, Mass General Brigham, Emory University, Boston Medical Center, Temple University, and University of California San Diego. No standardized precourse preparation or assigned reading was required before participation. Simulation fidelity (high-fidelity or low-fidelity) was determined using the local simulation resources available. High-fidelity simulation was defined as sessions conducted in a simulation center, which could incorporate physiologic monitors, and an environment closely resembling an ICU. By contrast, low-fidelity simulation was conducted in a classroom environment or conference room and used a manikin that was not capable of interactive responses. As depicted in Figure 1, trainees were still required to perform all bedside components of BD/DNC examination: opening the manikin's eyes to demonstrate testing pupillary and corneal reflexes, reviewing ventilator settings and titratable medications, and performing apnea testing by advancing an insufflation catheter to the level of the carina. In the low-fidelity model, the vital signs were displayed on an iPad “monitor” or whiteboard and updated by an embedded participant as the case unfolded. Static printouts of ventilator and pump screens were modified in real time to reflect trainee actions.

#### Participants

Eligible participants were neurology residents and neurocritical care fellows. Recruitment was by email invitation, which described study procedures and expectations. Participation was voluntary and not a requirement for residency or fellowship completion. Given that this was a pilot intervention, we did not conduct a power calculation. Each site investigator was asked to implement at least one simulation case, with all completed cases contributing to the study data set; some sites enrolled multiple participants. Owing to IRB-related delays at some centers, the experience of the first 6 enrolling institutions is reported here. These institutions' experiences informed final design of the curriculum because these pilots were completed before launch of the ongoing competency study.

#### Intervention

Participants completed the precourse survey and were assigned to one of 3 BD/DNC cases. Which cases were piloted was left to the discretion of the local site investigator. Simulations were delivered using either the high-fidelity or low-fidelity format, based on institutional availability. Each simulation began with a case review in which the patient's vital signs, medical history, home medications, laboratory results (including urine toxicology), neuroimaging, and MAR were available on request. After addressing potential confounders, trainees performed the clinical examination, evaluated the safety and appropriateness of the apnea test, conducted the apnea test, and ordered an ancillary test if indicated. Participants were observed by neurointensivists from the study team who provided detailed feedback on performance at the conclusion of the case. The debriefing approach was left to the discretion of each site. Participants then completed the postcourse survey.

#### Data Analysis

Data were collected using Qualtrics and analyzed using R (version 4.5.1). Participants' practice institution, clinical experience in BD/DNC determination, acceptability items, and ACGME milestone self-assessments were summarized using descriptive statistics. Presimulation and postsimulation confidence items were grouped into 4 domains: (1) Setup and Family Counseling, (2) Prerequisites, (3) Neurologic Examination, and (4) Apnea Test and Declaration of BD/DNC. For each domain, a per-participant composite score was calculated as the median of item responses with interquartile ranges (IQRs), given ordinal nature of data. Presimulation and postsimulation domain scores were compared using the Wilcoxon signed-rank test. To account for multiple comparisons across the 4 domains, *p* values were adjusted using the Benjamini-Hochberg false discovery rate procedure, with an adjusted *p* (*p*_*adj*_) ≤ 0.05 considered statistically significant. Between-group differences were examined by comparing domain-level change scores (post-pre) between high-fidelity and low-fidelity cohorts using the Mann-Whitney *U* test. Effect sizes for within-group and between-group comparisons were calculated using the Wilcoxon effect size (*r*).

### Ethical Approval and Trainee Consents

The study was determined to be exempt by the Institutional Review Board at each participating site. Consent to participate was voluntary and implied by survey completion.

### Data Availability

The data that support the findings of this study are available from the corresponding author on reasonable request.

## Results

A total of 19 participants across 6 institutions were enrolled in the pilot study. Most (15/19, 79%) had observed or participated in ≤5 BD/DNC determinations. Participants' institutions, level of training, training history, simulation fidelity, and previous clinical experience with BD/DNC are summarized in Table 2.

**Table 2:** Pilot Participants' Institutions, Simulation Fidelity, Level of Training, and Clinical Experience in Brain Death/Death by Neurologic Criteria (BD/DNC) Determination (N = 19)

Institution of participants, N (%)	
Emory	9 (47.4)
University of Maryland	5 (26.3)
Temple University	2 (10.5)
Massachusetts General Brigham	1 (5.3)
Boston Medical Center	1 (5.3)
University of California San Diego	1 (5.3)
Postgraduate year (PGY) of training	
PGY-2	2 (10.5)
PGY-3	2 (10.5)
PGY-4	14 (73.7)
Neurocritical care fellow	1 (5.3)
Sex of participants, N (%)	
Female	9 (47.4)
Male	10 (52.6)
Simulation fidelity, N (%)	
High	10 (52.6)
Low	9 (47.4)
Clinical experience in BD/DNC, N (%)	
Observed or participated in >5 BD/DNC determination	4 (21.1)
Personally performed^a^ >5 BD/DNC determination	1 (5.3)
Observed BD/DNC explained to the patient's family	18 (94.7)
Personally explained BD/DNC to the patient's family	5 (26.3)
Observed the communication of BD/DNC results to the patient's family	16 (84.2)
Personally communicated BD/DNC results to the family	5 (26.3)

a Performed is defined as doing the hands-on components of the examination with attending supervision.

### Confidence and Acceptability Outcomes

**Figure 2:**
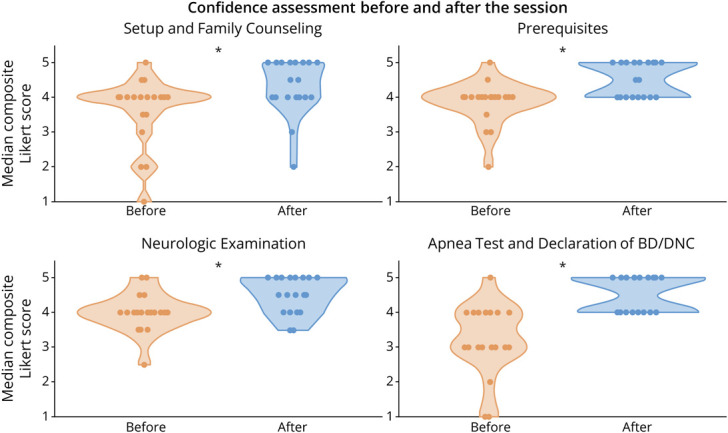
Trainee Confidence in Brain Death/Death by Neurologic Criteria (BD/DNC) Determination Confidence assessment across 4 domains of BD/DNC determination before and after simulation, which include the following: (1) “Setup and Family Counseling,” (2) “Prerequisites,” (3) “Neurologic Examination,” and (4) “Apnea Test and Declaration of BD/DNC.” Scores represent composite median Likert responses (5-point scale, 1 = strongly disagree, 5 = strongly agree). Asterisks indicate significance (adjusted *p* ≤ 0.05).

Composite confidence scores improved significantly across all 4 domains (all *p*_*adj*_ ≤ 0.05) (Figure 2, eTable 3). Median [IQR] pre/postintervention composite confidence scores increased from 4 [3.5–4] to 4.5 [4–5] (mean change 0.68, standard error of the mean [SE] 0.18; *p*_*adj*_ = 0.003; *r* = 0.82) for “Setup and Family Counseling,” from 4 [4–5] to 4.5 [4–5] (mean change 0.68, SE 0.13; *p*_*adj*_ = 0.002; *r* = 0.88) for “Prerequisites,” and from 4 [4–4] to 4.5 [4–5] (mean change 0.50, SE 0.14; *p*_*adj*_ = 0.004; *r* = 0.79) for “Neurologic Examination.” Notably, the largest improvement was observed in “Apnea Test and Declaration of BD/DNC,” which increased from 3 [3–4] to 5 [4–5] (mean change 1.32, SE 0.20; *p*_*adj*_ = 0.001; *r* = 0.88).

When comparing outcomes across fidelity groups, both high-fidelity and low-fidelity groups demonstrated similar improvements, with no statistically significant differences in the magnitude of change in composite confidence scores. Median [IQR] changes in composite confidence scores between high-fidelity and low-fidelity simulation were 0.75 [0–1] vs 0.5 [0.5–1] (mean difference 0.66, SE 0.18; *p*_*adj*_ = 0.965; *r* = 0.039) for “Setup and Family Counseling,” 0.5 [0.12–1] vs 1 [0–1] (mean difference 0.63, SE 0.13; *p*_*adj*_ = 0.965; *r* = 0.03) for “Prerequisites,” 1 [0–1] vs 0.5 [0–1] (mean difference 0.50, SE 0.14; *p*_*adj*_ = 0.965; *r* = 0.19) for “Neurologic Examination,” and 1 [0.25–1.75] vs 1 [1-1] (mean difference 1.05, SE 0.19; *p*_*adj*_ = 0.965; *r* = 0.02) for “Apnea Test and Declaration of BD/DNC” (eTable 4).

All participants (19/19, 100%) agreed or strongly agreed that the curriculum's objectives were clearly defined, met their educational expectations, and increased their confidence in BD/DNC determination. After completion of the curriculum, 13 of 19 participants (68%) self-assessed their ACGME milestone for BD/DNC determination as at least a Level 4 (“accurately performs death by neurologic criteria”).

## Discussion and Lessons Learned

In this multi-institutional pilot study, we leveraged the experience of a national group of simulation educators and BD/DNC experts to develop 3 BD/DNC cases that tested the participants' ability to navigate common confounders and pitfalls in the determination of BD/DNC, as well as collected validity evidence, established feasibility, and demonstrated Kirkpatrick Level 1 outcomes from participants. In our discussion, we first describe the pilot program's impact on trainees' confidence and self-assessed experience in BD/DNC, which may inform further research in this topic. Next, because this is one of the first nationally developed, deployed, and evaluated simulation curricula, we focus our lessons learned on our experiences in leading multi-institutional collaborative work, which may benefit other educators seeking to build multi-institutional simulation curricula.

### Discussion on the Pilot Findings

Several important themes about BD/DNC experience and training emerged from this pilot. First, in a group enriched with senior residents, less than a quarter of participants had substantial real-life experience in BD/DNC, which is consistent with previous research and emphasizes the need for targeted training.^4,9^ Second, participation in the simulation-based curriculum resulted in significant improvement in self-assessed confidence among multiple skill domains. Notably, the largest confidence gain was observed in the “Apnea Test and Declaration of BD/DNC” domain, which may reflect lower baseline familiarity with this high-stakes, infrequently performed test among neurology trainees as opposed to the “Neurologic Examination” domain. Furthermore, domains such as “Prerequisites” may be more conceptually understood before training, resulting in smaller observable gains. Although these results are subject to substantial caveats as described further, they support simulation as a potential intervention to support BD/DNC training.

### Lessons Learned for Multisite Collaboration

Our key lessons learned and solutions are summarized in Table 3. Additional discussion is provided in eTable 5.

**Table 3:** Challenges Faced in Multi-Institutional Simulation Design, Deployment, and Evaluation

Challenges	Specific examples and solutions from the brain death/death by neurologic criteria (BD/DNC) curriculum
National collaboration	Leadership with a shared interest in simulation, but without a clear project Meeting times needed to accommodate various time zones Need for a mechanism to share data Differences in institutional review board (IRB) expectations Need for content oversight
Divergent expectations for the trainees	Critical care knowledge Degree of independence in the apnea test Ability to use a checklist
Varied institutional protocols	BD/DNC protocols vary nationally
Lack of standardized simulation equipment and accessibility	Different access to simulation centers Differences in audio or video quality Inability to complete ocular vestibular reflex due to water incompatibility with simulator
Need to establish standardized case implementation and inter-rater reliability	Determination of a unified critical action checklist Standardization of course facilitation Avoiding prompting the participant to complete checklist items Establishing how to handle situation in which a participant does not know how to complete a critical action in BD/DNC determination, i.e., required to complete other critical actions (double jeopardy) Establishing how to score checklist items if the trainee went out of order (i.e., completed the apnea test before clinical examination) Establishing how to handle time/observation periods

### Establish a Multicenter Consortium

Conducting a multicenter simulation study necessitated the formation of a panel of neurology educators from different institutions with varying levels of expertise in simulation-based neurology education. To support this work, we established a dedicated consortium of simulation educators and BD/DNC experts—the Consortium for Research and Education in Simulation promoting Clinical Excellence in Neurology Training (CRESCENT). This collaboration was essential to address variations in institutional practices, trainee backgrounds, and available simulation resources. CRESCENT provided a platform, in the form of dedicated monthly meetings and an email listserv, for sharing expertise, feedback on the curriculum, and pilot experiences. Despite the clear advantage of bringing educators from across the country together, cross-country collaboration also had its associated challenges, as listed in Table 3.

### Navigate Divergent Expectations for Trainee Roles

Although resident expectations are governed by the ACGME milestones, programs do not have uniform expectations for what the trainee will be expected to independently manage. For example, during case design, one area of debate was the extent to which neurology residents should be expected to advise on management of critical care topics, such as adjustment of the ventilator and hemodynamic augmentation with vasopressors. While some educators advocated that comfort with critical care management was essential for ensuring appropriate apnea testing conditions, others considered these specifics outside the scope of neurology residency training. To balance these perspectives, the final curriculum for neurology residents assigned ventilator and hemodynamic management to an embedded intensivist. However, residents were expected to directly discuss safety of apnea testing with the embedded intensivist before proceeding with the apnea test.

### Recognize Variability in Institutional Protocols and Training Practices

As for many other topics in acute neurology, institutional protocols for BD/DNC remain heterogeneous, with differences in prerequisites, examination procedures, and examiner qualifications, despite the published consensus guidelines.^7,8^ Standardized training cases must, therefore, accommodate local practices while remaining broadly applicable. When institutional practices diverged from the BD/DNC guidelines, our group opted to maintain adherence to the national guidelines. For instance, not all institutions have required a 24-hour waiting period when the patient temperature drops below 35.5°C. However, our simulation case required the participant uphold the 24-hour delay before testing, consistent with the 2023 BD/DNC guidelines.

In areas where best practice has not been defined, the group selected the preferred approach through a consensus-based process. For example, in the BD/DNC cases, participants must select a method for apnea testing. The BD/DNC guidelines offer 4 acceptable practices: tracheal insufflation, flow-inflating resuscitation bag with a T-piece, positive end-expiratory pressure (PEEP) valve, and continuous positive airway pressure (CPAP) on the ventilator. Although studies suggest that CPAP/PEEP-based apnea testing may improve oxygenation and reduce hypoxemia compared with tracheal insufflation, no evidence has demonstrated superiority of any method in confirming BD/DNC.^20-22^ Our consortium selected tracheal insufflation as the strategy that would be taught in the curriculum. This agreement was reached by the group because this method was most commonly used throughout the consortium and was readily available at all sites. Standardizing this aspect of the test reduced the potential for unforeseen protocol deviations, as well as obviated the need to create separate checklists based on the trainee's approach.

### Embrace the Simulation Fidelity Available

The pilot reinforced the importance of designing simulations that closely replicate real-life clinical scenarios, which is a crucial component of content validity. We agreed that trainees should have access to the same resources they would use in clinical practice, such as institutional protocols and reference materials. This has been shown to enhance skill transfer from simulation to practice,^23^ reduce extraneous cognitive load,^24^ and improve deliberate practice.^25^ To accommodate centers without high-fidelity simulation equipment, supplementary materials—including printed baseline data and ventilator settings—were developed to enable participation. It is important to note that participants in both high-fidelity and low-fidelity groups demonstrated comparable gains in confidence across all domains, with no significant differences in the magnitude of improvement. Our results suggest that effective BD/DNC training can be achieved in resource-limited settings, where high-fidelity equipment is not available.

### Modification of Materials Based on Feedback

The pilot highlighted the need for consistent facilitation across institutions. An evolving reference document was created to standardize facilitator responses to common trainee questions (eAppendix 3). Furthermore, trainees were encouraged to verbalize each step during their BD/DNC assessment as we recognized that this facilitated more consistent observation, feedback, and deliberate practice.

## Limitations

This study has several limitations. For most institutions, participation in the pilot was voluntary, introducing self-selection bias and limiting generalizability. Indeed, a substantial proportion of trainees (n = 8/18) pursued inpatient-focused subspecialties. Because these residents are more likely to encounter BD/DNC in their future clinical practice, they may represent a group that is more invested in the training than residents that are unlikely to encounter BD/DNC in their future clinical practice. Second, recruitment varied across sites and was lower than anticipated at several institutions, in part due to delays in local IRB approval and differences in site readiness. Nearly half of the participants were recruited from a single site, which may also limit generalizability and raise the possibility of site-level clustering. Participants from the same institution may share similar training experiences, institutional practices, and exposure to BD/DNC, potentially influencing both baseline confidence and responsiveness to the curriculum. One site was overrepresented because BD/DNC training was a program priority, with mandatory participation for all PGY-4 residents. Notably, this site exclusively used low-fidelity simulation, providing an opportunity to evaluate feasibility in resource-limited settings. Although no differences in Kirkpatrick Level 1 outcomes were observed, this finding should be interpreted as preliminary because the study was not powered to detect small differences between fidelity groups. These results should, therefore, be considered hypothesis-generating and warrant further evaluation in larger, adequately powered studies.

Third, we did not assess knowledge acquisition or performance skills because this pilot was designed primarily to evaluate feasibility and acceptance by participants. However, this pilot paved the way for a currently enrolling study that aims to evaluate subsequent behavior change and skill retention in BD/DNC determination using SBML. Fourth, although training level may influence baseline confidence and learning gains, our sample was highly imbalanced (14 participants were postgraduate year 4 trainees), and adjustment for training level would have produced unstable estimates. We could not perform this analysis here but hope to examine this as recruitment continues in the ongoing prospective study. Fifth, we did not collect baseline ACGME milestone self-assessment data, which limits our ability to evaluate preintervention to postintervention change and may be influenced by variability in training year. Sixth, although 3 distinct cases were designed to capture difference challenges in BD/DNC determination, the sample size limited our ability to analyze confidence changes at the level of individual cases. As such, findings should be interpreted at the aggregate level across domains rather than case-specific effects. Finally, previous clinical exposure to BD/DNC determination was collected in a binary format (≤5 vs >5), which limited our ability to examine how more granular differences in experience may have influenced baseline confidence or learning gains.

## Closing Remarks

This pilot study demonstrated the feasibility of implementing a consensus-based BD/DNC simulation curriculum across multiple institutions, although adoption and recruitment varied across sites. Key curricular features—including simulation realism, standardized facilitation, and flexibility to accommodate local resources—supported implementation across diverse settings. However, implementation challenges, including differences in site readiness, recruitment, and available resources, highlight important consideration for broader dissemination. While improvements in learner confidence were observed, these findings are preliminary and warrant evaluation in larger, more diverse cohorts. Lessons learned from this pilot can inform future research in BD/DNC education and multi-institutional simulation efforts.

## Appendix Coinvestigators

**Table:**

Name	Location	Role	Contribution
Galina Gheihman	Department of Neurology, Harvard Medical School, Mass General Brigham, Boston, MA	Educational researcher	Case implementation; manuscript
Shivani Ghoshal	Columbia University Vagelos College of Physicians and Surgeons, New York, NY	Academic neurointensivist	Case design and refinement
Hannah Kirsch	Stanford University School of Medicine, San Francisco, CA	Academic neurointensivist	Case design and refinement
Jon Rosenberg	Department of Neurology and Neurosurgery, Westchester Medical Center, Valhalla, NY	Academic neurointensivist	Case design and refinement

## Glossary

ACGME: Accreditation Council for Graduate Medical Education
BD/DNC: brain death/death by neurologic criteria
CPAP: continuous positive airway pressure
IQR: interquartile range
PEEP: positive end-expiratory pressure
SBML: simulation-based mastery learning
